# Quantitative abdominal sodium MRI combined with 32‐channel proton pTx MRI at 7 Tesla in a large field‐of‐view

**DOI:** 10.1002/mrm.30605

**Published:** 2025-06-22

**Authors:** Anna K. Scheipers, Johannes A. Grimm, Jana Losch, Stephan Orzada, Thomas M. Fiedler, Armin M. Nagel, Sebastian Schmitter, Mark E. Ladd, Tanja Platt

**Affiliations:** ^1^ Medical Physics in Radiology German Cancer Research Center (DKFZ) Heidelberg Heidelberg Germany; ^2^ Faculty of Physics and Astronomy Heidelberg University Heidelberg Germany; ^3^ University Hospital Erlangen, Institute of Radiology University of Erlangen‐Nuremberg (FAU) Erlangen Germany; ^4^ Physikalisch‐Technische Bundesanstalt (PTB) Braunschweig and Berlin Germany; ^5^ Faculty of Medicine Heidelberg University Heidelberg Germany

**Keywords:** high‐field MRI, quantitative imaging, 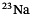, sodium, parallel transmit (pTx), whole body imaging

## Abstract

**Purpose:**

To combine large field‐of‐view abdominal 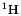 and quantitative sodium (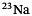) MRI in the same position at 7 T to enable the quantification of the tissue sodium concentration via 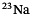 MRI and the anatomical assignment in 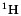 images without repositioning in several tissues and organs at once.

**Methods:**

A custom‐built 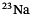 birdcage coil and reference vial setup together with a 32‐channel 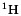 pTx array were employed at 7 T to allow dual‐nuclei MRI in the same position for a field‐of‐view (FOV) of $(400\times 400\times 400)\;{\text{mm}}^{3}$. $T_{1}$ relaxation effects in the reference vials and phantom were corrected, $B_{1}^{+}$ maps were measured, and $B_{1}^{-}$ maps were simulated to correct the acquired 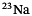 data in post‐processing. These corrections were evaluated in a phantom and then applied in vivo in three healthy volunteers.

**Results:**

In the phantom, it was demonstrated that proton and quantitative sodium MR images share the same large FOV. Phantom measurements showed an improved sodium concentration accuracy after the performed corrections. Large FOV 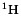 and quantitative 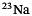 in vivo MRI was demonstrated in three healthy volunteers.

**Conclusion:**

This work shows the feasibility of combined 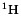 and quantitative 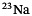 imaging at 7 T in a large FOV both under free breathing in ≤47min, laying the ground work for an accurate evaluation of the tissue sodium concentration in several organs at once.

## 1 INTRODUCTION

Sodium ions play an important role in many metabolic processes in the human body. In healthy cells, the sodium‐potassium ATPase maintains the resting potential of the cell,^1^, ^2^ with a gradient between the intra‐ and extracellular sodium concentration.^3^ The tissue sodium concentration (TSC) is the volume‐weighted concentration average of both these compartments^4^ and can be quantified using sodium (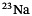) MRI.^1^, ^2^, ^5^, ^6^, ^7^ The TSC is sensitive to changes in cell vitality and viability and can thus be a potential biomarker for a wide variety of applications ranging from lumbar back pain^8^ to heart disease^9^ to chronic kidney disease.^10^ To obtain a quantitative 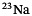 image, the signal of the MR image is calibrated using the known sodium concentrations in internal^11^, ^12^, ^13^ or external references.^14^, ^15^, ^16^

Sodium MRI is connected to several challenges complicating the image acquisition such as a low MR sensitivity and low in vivo ${\text{Na}}^{+}$ concentrations, leading to a lower signal‐to‐noise ratio (SNR) in vivo compared to hydrogen.^17^ As the SNR is expected to increase at least linearly with the magnetic field strength $B_{0}$ for 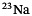,^17^ an adequate SNR can be obtained in a clinically feasible measurement time at 7 T.^18^ Nevertheless, acquisition times for sodium body MRI in a large field‐of‐view (FOV) are regularly exceeding 10min in measurement time, which may lead to motion artifacts and thus a decrease in image quality.^1^ Furthermore, the fast bi‐exponential transverse relaxation times require pulse sequences with short echo times, posing high demands on the hardware.^19^ As sodium images commonly acquired in vivo typically suffer from a low spatial resolution due to the low in vivo SNR, it is less suited for segmentation than proton MRI. Registration of 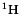 and 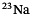 images acquired in two different examinations can be quite challenging, especially in the torso, due to changes within the organs as a result of the different posture within a different coil and/or physiological differences between the two scans, e.g., due to digestive processes, rectal and bladder filling, etc. Thus, acquiring both images in the same examination is favorable, as it reduces the potential error of image registration, resulting in more reliable segmentation. 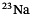 MRI, especially at 7 T, is oftentimes not combined with 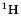 MRI,^3^ as many of the RF antenna designs are 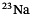 only.^14^, ^20^, ^21^, ^22^ In the last years, more dual‐nuclei coils were designed,^16^, ^23^, ^24^ recognizing the importance of anatomical information for localization or segmentation purposes. Such a dual‐nuclei coil setup in the limited space within the bore opening becomes even more challenging with quantitative MRI if external reference vials need to be positioned in the field‐of‐view.

$B_{1}$ inhomogeneities within the field‐of‐view need to be considered in case of quantitative 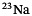 MRI. Here, larger $B_{1}$ inhomogeneities lead to stronger influences on the acquired 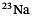 data and Lommen et al.^25^ showed that $B_{1}^{+}$ corrections are more prone to errors with such larger variations. One advantage of our 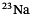 RF coil is that the variations in the fields are not as pronounced.^21^ The often employed assumption that $B_{1}^{+}$ = $B_{1}^{-}$ is not valid for the 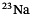 coil used, as the hardware used for the transmit and the receive part of the custom‐built birdcage coil differ.^21^ Since the acquisition of in vivo $B_{1}^{-}$ maps is not possible with this RF coil design without separate channels for transmission^26^ or a birdcage for reception,^27^ $B_{1}^{-}$ maps were simulated for each of the measured setups.


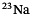
 MRI has mostly been focused on a single region of the body such as the kidney,^11^, ^14^, ^23^ the heart^23^, ^24^ or the intervertebral discs^13^, ^28^ applying local RF coils, customized for one or two specific target anatomies, which often show a limited anatomic coverage and penetration depth, especially in the case of surface coils. The evaluation of more than the target organ is thus often not possible in these cases.

A large field of view is required for torso MRI, especially if several organs are of interest. Boehmert et al.^23^ discuss the development of an RF coil for 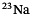 and 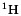 MRI of the heart or kidneys to investigate the cardiorenal syndrome, a disorder where the dysfunction of the kidneys induces a dysfunction of the heart or vice versa.^29^ This is one example where the simultaneous acquisition in both organs would be preferable over acquiring two separate scans to save scan time and gain a better understanding of the interdependence of both organs.

The setup presented here enables obtaining the apparent tissue sodium concentration (aTSC) of organs, e.g., in the whole upper torso (heart, lungs, and abdomen), to compare the quantitative sodium concentrations between different volunteers. As suggested by Stobbe and Beaulieu,^30^ the term aTSC was used for the determined sodium concentration values to consider unaccounted influences. In the presented work, these are not exactly known signal losses from relaxation and pulse sequence characteristics, as well as influences due to unperformed corrections or unknown inaccuracies in the performed corrections. In the future, the knowledge of the aTSC in healthy volunteers may help to differentiate normal levels of physiological change from changes due to diseases. Being able to access the aTSC in several organs at once means that organ‐specific scans (e.g., a kidney‐only scan) can be omitted in most cases while still benefiting from the additional information of 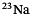 MRI in several organs and tissues. Knowledge of the aTSC in a large FOV could, e.g., also help to monitor treatment success of chemotherapy patients with whole body metastases.^22^ It also allows investigation of body regions that have not been examined yet, such as the pancreas and stomach, without performing an additional scan.

While 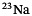 MRI benefits from the increased SNR at ultra‐high field strengths, 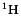 MRI is becoming more challenging, especially in the torso. The operational frequency of proton MRI at $B_{0}$ = 7 T is 297.1 MHz, leading to a wavelength in tissue of about 11 cm, 13 cm, and 42 cm for water, muscle and fat, respectively,^31^ which causes signal dropouts in the transmit field distribution. To improve the homogeneity, parallel transmit (pTx)^32^ and time interleaved acquisition of modes (TIAMO)^33^ will be used here. As the operational frequency of sodium is about 78.6 MHz at $B_{0}$ = 7 T, which is comparable to 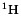 MRI at $B_{0}$ = 1.5 T, the standing waves and interferences do not significantly impact the image quality^18^, ^34^ and pTx optimization is not required.

The goal of the presented work is to combine large field‐of‐view abdominal 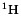 and quantitative 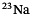 MRI in the same position at 7 T for the first time. Here, 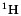 MRI gives the advantage of anatomical images and $B_{0}$ shimming capabilities and the corrected quantitative 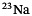 MRI provides insights into physiology.

## 2 METHODS

### 2.1 Ethics approval

In vivo studies were approved by the ethics committee of the Medical Faculty Heidelberg, Germany (S‐154/2014). Written informed consent was obtained from all participants included in the measurements prior to their examinations.

### 2.2 MR system

The measurements for this study were performed on a 7 T whole‐body MR research system (MAGNETOM 7T, Siemens HealthCare GmbH, Erlangen, Germany) at the German Cancer Research Center (DKFZ) in Heidelberg.

The scanner supports multinuclear applications using a broadband amplifier within an RF frequency range from 14 MHz to 130 MHz, allowing measurements of X‐nuclei such as 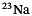 with one transmit channel.

### 2.3 RF coils

#### 2.3.1 Sodium RF coil

**FIGURE 1:**
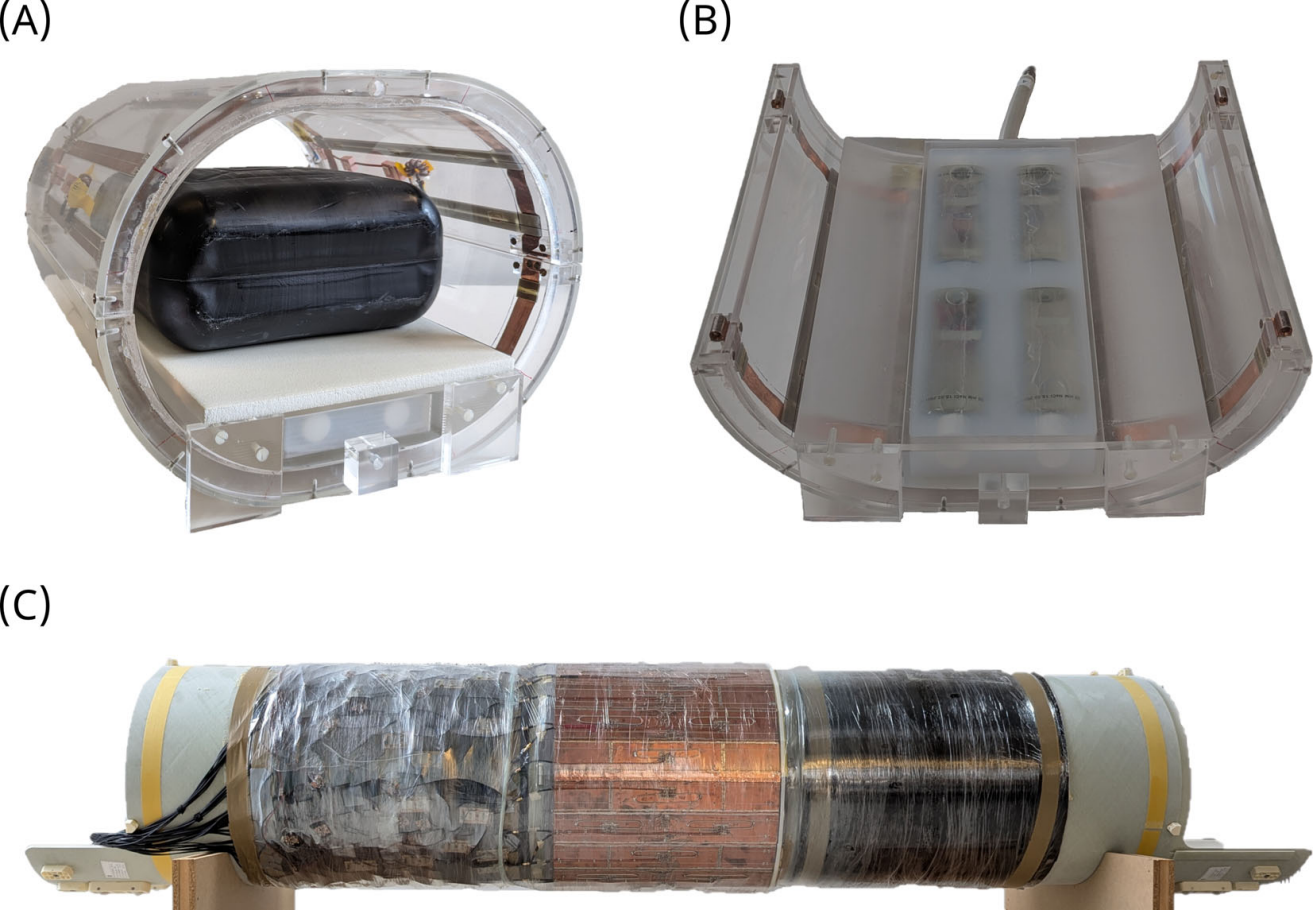
Picture of (A) the 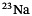 RF coil containing the reference vial setup and phantom, (B) the reference vial setup within the lower part of the 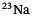 RF coil, showing the reference vial placement, and (C) the 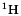 RF coil outside of the scanner bore.

The applied custom‐built oval‐shaped birdcage coil (Figure 1A,B) is a four‐port birdcage coil, which acquires four receive signals during reception separately.^21^ It has a large field‐of‐view of (400 mm)^3^ and homogeneous transmit (Tx) and receive (Rx) field distributions. For in vivo measurements, the arms are positioned above the head outside of the coil.

#### 2.3.2 Proton RF setup

For the 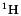 measurements, a custom‐built 32‐channel pTx body coil^35^ was used for transmission as well as reception (Figure 1C). The 32 antenna elements are placed in three rings between the bore liner and the gradient coil. This configuration allows the simultaneous use of the local 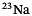 coil as shown in Figure 1A and the remote 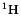 coil.

### 2.4 Reference vial setup

For the concentration determination, a fixed reference vial setup was developed. This setup was placed in a fixed position below the subject to minimize the impact of respiratory motion on the vial placement and thus the results in the vials. Within the interchangeable compartment, which works like a drawer, 4 oval‐shaped reference vials (Rixius AG, Mannheim, Germany) filled with demineralized water and known concentrations of sodium are embedded in a mixture of 35 mm NaCl, 2% agarose and demineralized water to reduce susceptibility artifacts and to improve $B_{0}$ shimming capabilities. The bottles contain a nominal volume of 250 mL with a length of 59.8 mm, a width of 38 mm, and a height of 169 mm. For phantom measurements, vials containing 20 mm, 30 mm, 40 mm and 50 mm NaCl and demineralized water were used. For in vivo measurements, vials with sodium concentrations of 20 mm, 60 mm, 100 mm, 140 mm were used to span the expected range of aTSC values within the torso. The compartment for the reference vials was designed in‐house, along with the drawer housing that serves as the volunteer's laying surface. Both are manufactured out of acrylic glass and displayed in Figure 1A underneath the phantom and in Figure 1B within the lower part of the 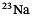 RF coil. Overall, the structure takes up a maximal height of 52 mm within the coil.

Since the reference vials are always positioned at the same location in the coil, the corresponding masks can be reused in every examination.

### 2.5 Phantom

A 10 L canister (hünersdorff GmbH, Ludwigsburg, Germany) filled with 35 mm of NaCl and demineralized water was used as a homogeneous phantom to validate the measurement and correction routines before conducting volunteer measurements. It is approximately 30.6 cm long, 16 cm wide, and 27 cm high with rounded edges and corners (Figure 1A).

### 2.6 EM simulations

The setup with the reference vials was modeled in the electromagnetic field simulation software CST Studio Suite 2020/2021 (Dassault Systèmes, Vélizy‐Villacoublay, France). For this, the dielectric properties of the contents of the reference vial setup and the phantom were measured using the Dielectric Assessment Kit (DAK) with the DAK‐12 Probe Geometry, the DAK software (Schmid & Partner Engineering AG (SPEAG), Zurich, Switzerland, Version: DAK 2.0.0.462) and a E5061B ENA Series Network Analyzer (Agilent Technologies, Santa Clara, CA, USA) for a frequency of ν = 79 MHz. The rounded values presented in Table 1 were used for the electromagnetic field simulations of the receive field maps.

**TABLE 1:** Dielectric properties of the fluids and gel in the reference vial setup and phantom for a frequency of ν=79 MHz. $\varepsilon^{\prime }$ is the real part of relative permittivity, σ is the conductivity.

[${\text{Na}}^{+}$] (mmol L 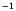 )	Agar conc. (%)	$\varepsilon^{\prime }$	σ (S m 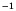 )
20	0	79	0.22
30	0	79	0.33
35	0	79	0.38
35	2	79	0.39
40	0	79	0.44
50	0	79	0.54
60	0	79	0.64
100	0	79	1.00
140	0	79	1.39

From the simulated radio frequency fields of the four receive channels, the $B_{1}^{-}$ field distributions were extracted and exported. Subsequently, a sum‐of‐squares combination (SOS) of the $B_{1}^{-}$ fields of the four Rx channels was calculated and applied for $B_{1}^{-}$ corrections.^12^

#### 2.6.1 Phantom simulation

**FIGURE 2:**
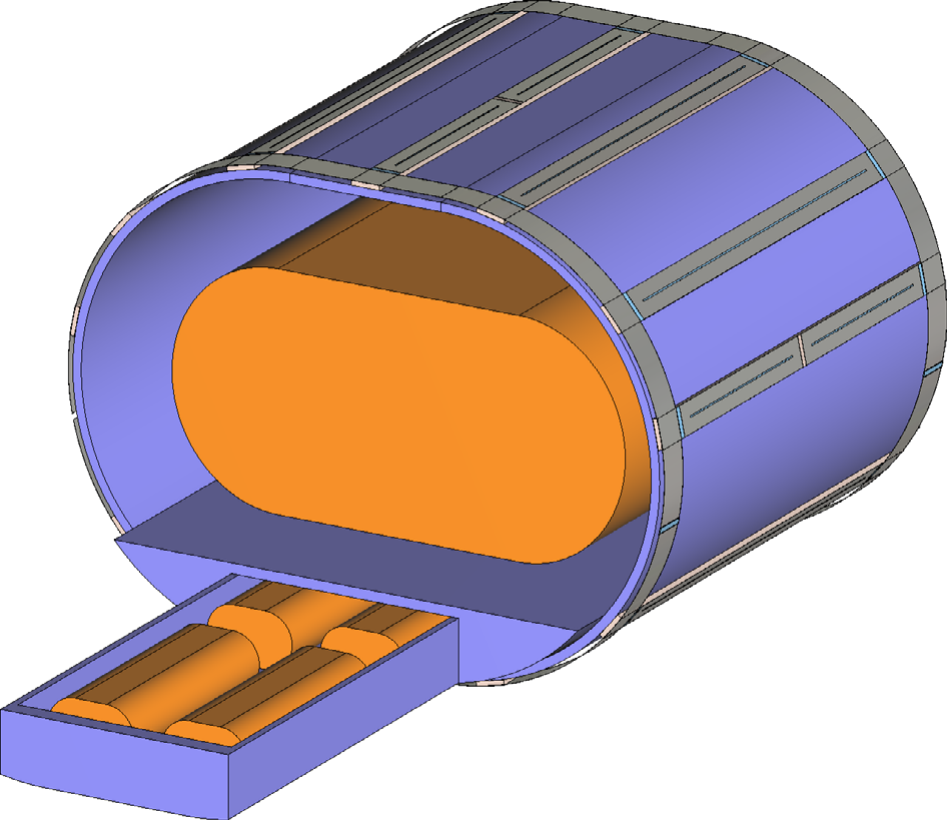
Simulation model of the reference vials and phantom in the 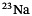 body coil for the $B_{1}^{-}$ field determination. A 3D view with the calibration drawer outside of the setup for demonstration purposes is shown.

The oval‐shaped reference vials were approximated as two cylinders with a diameter of 38 mm and a length of 152 mm, and a cuboid connecting them with a width of 22 mm. The phantom was constructed from a cuboid with a width of 170 mm and a length of 270 mm between two ellipsoidal cylinders of the same length, a height of 16 cm and a width of 15 cm (Figure 2). The corresponding dielectric properties were used in each of the different compartments according to Table 1.

#### 2.6.2 Human model simulation

A male (“Duke”, 34 years, 174 cm, 70 kg, tissue resolution 2×2×2 mm) and a female (“Ella”, 26 years, 160 cm, 58 kg, tissue resolution 2×2×2 mm) anatomical body model^36^ with tissue parameters from Gabriel et al.^31^ were placed inside the coil on top of the reference vial setup. The kidney region was positioned centrally inside the coil. The human voxel models were scaled isometrically to fit the volunteer's anatomy, creating a subject‐specific simulation. Ella was used both unscaled and scaled up by 12%, while Duke was scaled up by 3%.

### 2.7 Phantom measurements

To determine the $B_{0}$ shimming volume, 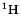 localizer images were acquired. Subsequently, $B_{0}$ shimming of the whole phantom and vial setup was performed. A 3D 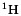 GRE sequence with radial phase‐encoded (RPE) acquisition (GRE‐RPE)^37^ that has been adapted to 7 T^38^ was applied utilizing TIAMO^33^ by applying two modes optimized for a torso‐simulating polyvinylpyrrolidone phantom mimicking the dielectric tissue parameters of the human body.^21^ The GRE‐RPE sequence was used in phantom and in vivo measurements, as it allows for binning into different respiratory states in future human studies.

Prior to the sodium measurement, a calibration to determine the 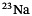 reference voltage $U_{\text{ref}}$ was performed. $U_{\text{ref}}$ is defined as the voltage of a rectangular excitation pulse of length τ = 1 ms that is needed to invert the equilibrium magnetization ($\alpha =180^{\circ }$). The phantom reference voltage was $U_{\text{ref}}=$ 1267 V. The pulse voltage for the applied $61^{\circ }$ rectangular pulse with a pulse duration of 1.8 ms is approximately 239 V, and the applied RF power during the pulse is approximately 1.14 kW.

For 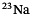 MRI, a 3D density‐adapted projection reconstruction sequence (DA‐3DPR)^39^ following a golden angle projection scheme^40^ was employed for both, the quantitative image and the transmit field map. The latter was measured with the dual flip angle method^41^ implemented in a pulse sequence with alternating excitation.^42^ The resolution of the $B_{1}^{+}$ maps was chosen to be ${\left(\Delta x\right)}^{3}$ = (20 mm)^3^ to reduce measurement time, which is sufficient as $B_{1}$ maps usually do not change fast on small spatial scales.^21^ The acquisition parameters are listed in Table 2. For 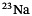 MRI, the utilized spin density weighted projection reconstruction sequence can achieve ultrashort $T_{E}$ (UTE) times, which limits the influence of $T_{2}^{*}$ on the quantification. To reduce the influence of the $T_{1}$ decay, a long repetition time ($T_{R}$ = 150 ms) was chosen. Two points in k‐space center were acquired, which could in the future be used for retrospective respiratory sorting of the 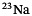 data.^21^

**TABLE 2:** Acquisition parameters and reconstruction settings for the different employed sequences for the phantom and in vivo measurements.

	Quantitative 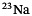 image	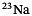 $B_{1}^{+}$ map (alternating excitation)	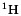 3D GRE‐RPE static pTx
$T_{E}$ (ms)	1	1.65	2.04
$T_{R}$ (ms)	150	106/168 (alternating)	4.23
Nominal FA (°)	61	45/90 (alternating)	11
Acquired isotropic nominal resolution (mm)	5	20	1.25
Projections	10 000	1020	—
Number of RPE lines	—	—	1536
Radial samples	256	256	—
Acquisition time (min:s)	25:00	4:39	17:20
Pulse duration (ms)	1.8	3	1
Readout duration (ms)	5	3.33	—
Bandwidth (Hz/Px)	—	—	1042
Acquired data points in k‐space center	2	2	—
$B_{1}^{+}$ shim mode	—	—	TIAMO (2 modes)
Zerofilling factor	2	8	—
Filter	Hamming	Gauss (σ = 20 mm)	—
reconstructed FOV (mm^3^)	400×400×400	400×400×400	400×400×400

### 2.8 In vivo measurements

Three healthy volunteers were measured after fasting for at least 9 h (2 female, 1 male, age: 35 years, 26 years and 22 years, weight: 85 kg, 56 kg, and 85 kg, height: 180 cm, 160 cm, and 184 cm). All sequences were acquired under free breathing without triggering and in a feet‐first supine position, with arms and hands placed above the head. The volunteers were positioned inside the 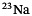 coil in such a way that the kidneys were approximately at the center of the coil along the z‐axis. 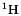 localizer images were obtained to verify the volunteer's position within the RF coil before starting the measurements.

The same measurement routine as described for the phantom measurements was used. The range of the in vivo reference voltage $U_{\text{ref}}$ was 1304 V to 1379 V. The applied RF power during the pulse utilized here is approximately 1.21 kW to 1.35 kW.

$B_{0}$ shimming of the whole body was performed. For 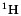 imaging, the specific absorption rate (SAR) was limited by limiting the input power per channel. Therefore, a custom safety supervision system as described by Fiedler et al.^43^ was used, which automatically shuts off if any channel exceeds the 10 s or 6min power limit for the torso, which was predefined according to EM simulations.

The acquisition parameters are listed in Table 2. Including all sequences, the total acquisition time was 46min and 59 s.

### 2.9 Postprocessing


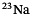
 images were reconstructed with a Nonuniform Fast Fourier Transform (NUFFT)^44^ employing the settings summarized in Table 2 to reconstruct matrix sizes of 160×160×160. 3D 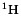 images were reconstructed using iterative SENSE^45^ and were interpolated to match the 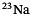 image resolution.

Since the 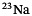 $T_{1}$ time in the reference vials and phantom is longer than in tissue but known, 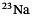 $T_{1}$ relaxation effects in these compartments were corrected according to the FLASH equation.^46^ For $T_{1,\mathrm{NaCl}}$ = 55 ms^47^ and $T_{1,\mathrm{kidney}}$ = 34 ms,^3^ the correction factor becomes 0.965 and 0.994 for a nominal flip angle of $61^{\circ }$, respectively. The quantitative image is then still influenced by inhomogeneities in transmit ($B_{1}^{+}$) and receive ($B_{1}^{-}$) fields of the utilized coil,^1^ for which the corrections are described below.

Datasets for $B_{1}^{+}$ maps were Gauss‐filtered (σ = 20 mm), while data for the quantitative 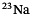 image were filtered with a Hamming filter. Quantitative images were zerofilled with a zerofilling factor (ZF) of 2, and $B_{1}^{+}$ maps were zerofilled with ZF = 8 to have the same matrix size and image resolution (160^3^ and (2.5 mm)^3^, respectively). An SOS combination of the 4 receive channel signals of the 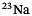 RF coil was performed.

The acquired nominal resolution of the quantitative 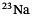 images is (5 mm)^3^. To estimate the effective resolution, the point spread function was simulated, which resulted in a FWHM of 1.7 for the DA‐3DPR before being further widened by a Hamming filter to a FWHM of 2.1, leading to an effective resolution of about (10.5 mm)^3^ for the quantitative 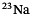 images.

To correct for $B_{1}$ inhomogeneities in the quantitative 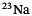 image, transmit field maps were additionally measured and receive field maps of the 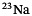 RF coil were simulated as described in the EM simulations section. The $B_{1}^{+}$ correction was performed with the sine of the flip angle map, while $B_{1}^{-}$ enters linearly.

### 2.10 Concentration determination

Quantitative 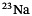 images were calculated with a semi‐automated segmentation tool, self‐developed in Matlab (The MathWorks Inc., Natick, MA, USA, Version R2024b) using the known concentration in the reference vials and interpolation based on a linear regression fit. 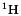 images and knowledge of the reproducible vial positioning were used to create a volume of interest (VOI) as a binary mask for each reference vial, which was then reused for all volunteers. The masks had a size of 5984 voxels per reference vial, corresponding to 93.5 mL. Detailed information on mask size can be found in Figure S1. They were positioned in the central part of the vials to reduce partial volume effects.

For the phantom data, a mask that only contains the phantom was automatically created in MATLAB based on the MR signal and by excluding the leftover outer parts of the reference vials from the mask. The outermost pixels of the phantom in each direction were excluded to ensure that no partially volumed pixels were used for the concentration determination. To assess local homogeneity within the phantom and the accuracy of 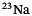 quantification, two smaller cuboid VOIs—one placed in the peripheral part of the phantom and one in a central position—were evaluated.

Since the concentration means in different VOI vary and the standard deviation is dependent on concentration levels, the coefficient of variation (CV) instead of the standard deviation is used to compare the influence of the different correction approaches on the homogeneity of the apparent sodium concentration (aSC) in the phantom after each correction step.

## 3 RESULTS

### 3.1 Phantom measurements

**FIGURE 3:**
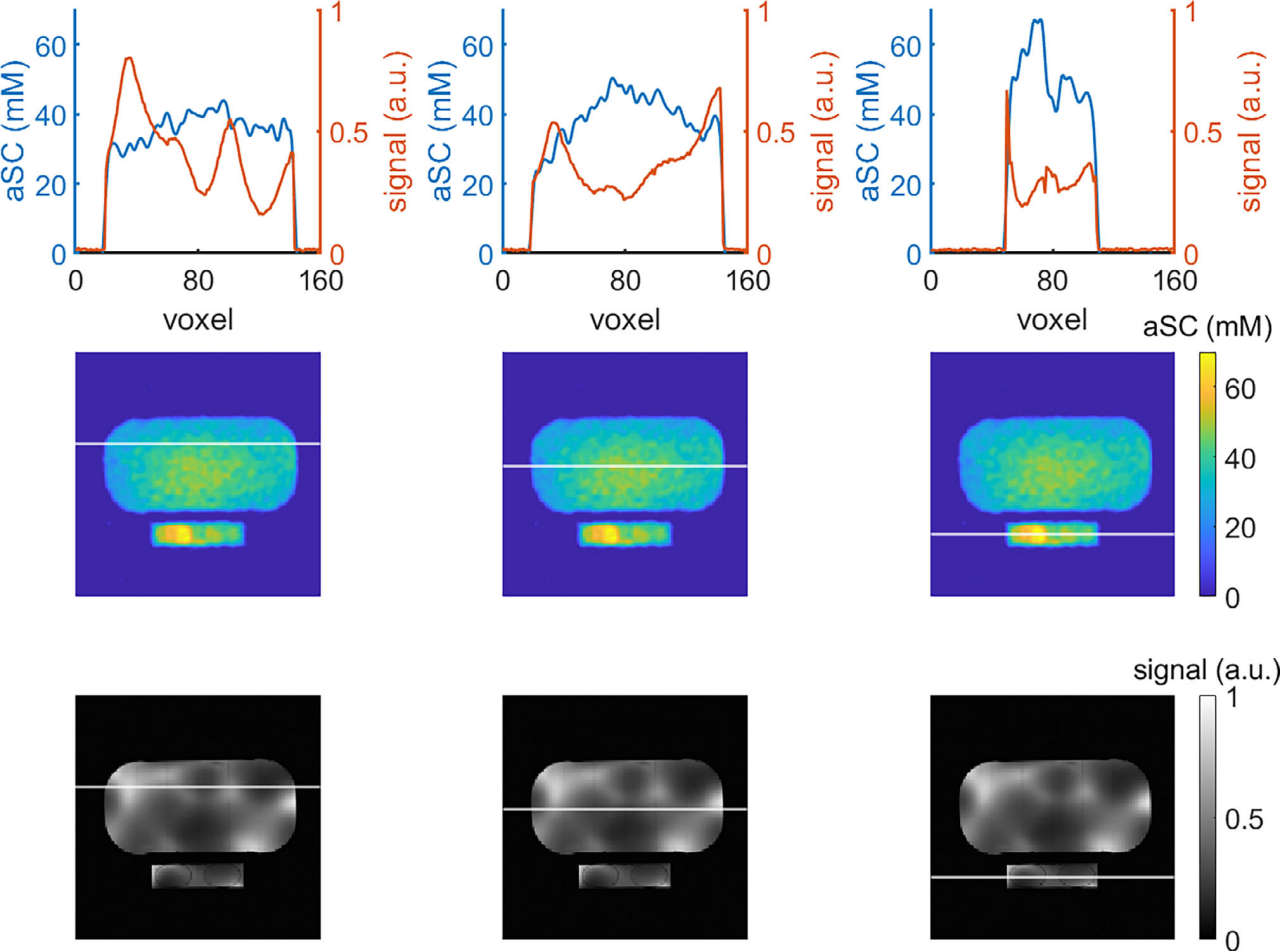
Alignment of 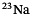 and 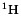 images in the phantom for the left‐right direction in a transversal slice. The line plot in the first row displays the values in the uncorrected 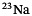 (blue line) and 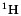 (orange line) MRI along the white line in the second and third row, respectively. For each plotted line through the phantom, the border of the phantom and reference vials in the 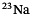 image correspond well to those in the 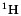 image. In the third column, where the values depicted are only in the reference vials, the sodium images show, as expected, a clear difference between the two reference vials with differing concentrations, despite remaining inhomogeneities in the uncorrected image.

Both, the 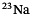 and 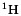 MR data match in position (see Figures 3 and  S2–S3), which allowed overlaying the images without image registration.

**FIGURE 4:**
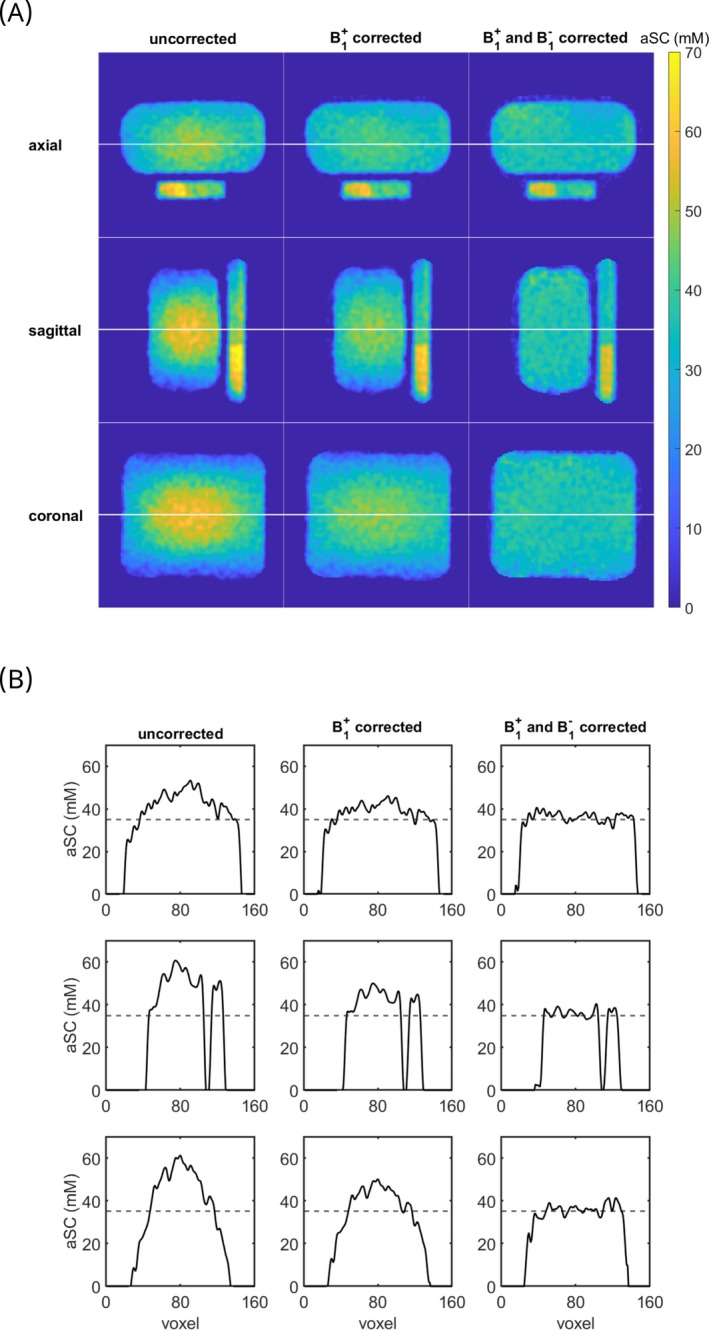
Influence of the applied corrections on the apparent sodium concentration (aSC) of a phantom filled with a homogeneous concentration of NaCl solution. The first column of (A) shows the uncorrected data, the second column the $B_{1}^{+}$ corrected data, and the last column the $B_{1}^{+}$ and $B_{1}^{-}$ corrected data. The plotted slices for the first two rows were chosen slightly off‐center to show the concentration difference within the reference vials. (B) shows line plots through a central slice in each of the in (A) plotted directions before corrections (first column), after the $B_{1}^{+}$ correction (second column), and after both $B_{1}^{+}$ and $B_{1}^{-}$ corrections (third column). The dotted line depicts the ground truth of 35 mm.

**FIGURE 5:**
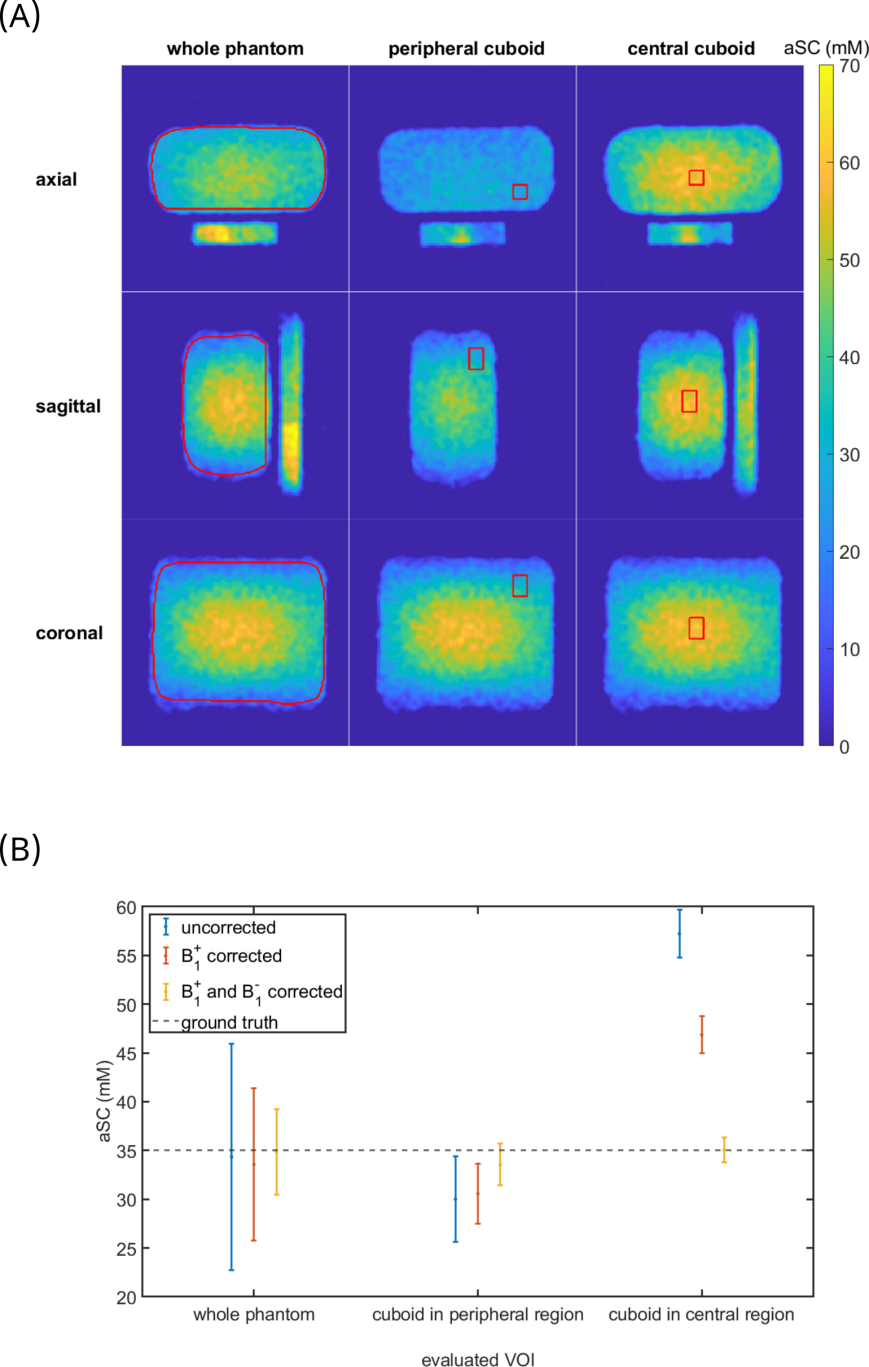
(A) shows the placement of the VOIs within the phantom. The VOI for the whole phantom (left) is automatically determined from the measured $B_{1}^{+}$ map, the simulated $B_{1}^{-}$ map, and the placement of the reference vials. The slices in the first two rows were chosen slightly off‐center to depict the concentration difference within the reference vials. The placement of the additionally evaluated cuboid VOI in the peripheral (middle) and central (right) part of the phantom is shown for different slices in the axial and sagittal views, but in the same coronal slice (last row). (B) shows the comparison of the determined aSC within the phantom before corrections (blue), after the $B_{1}^{+}$ correction (red), and after both $B_{1}^{+}$ and $B_{1}^{-}$ corrections (yellow) for the three in (A) shown VOI.

**FIGURE 6:**
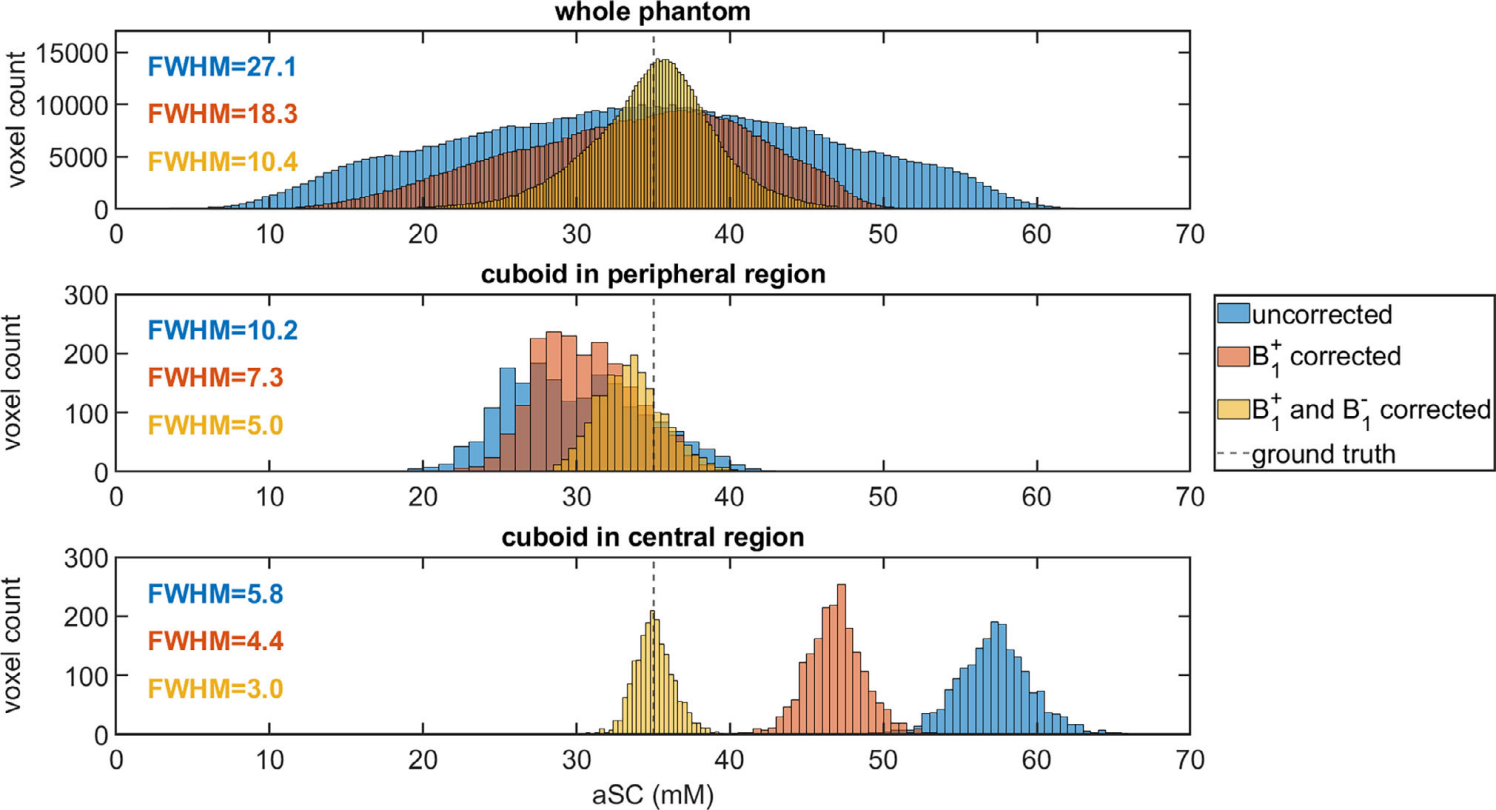
Histogram of the determined aSC within the phantom before corrections (blue), after the $B_{1}^{+}$ correction (red), and after both $B_{1}^{+}$ and $B_{1}^{-}$ correction (yellow) for the three in Figure 5A shown VOIs. The bin width was chosen according to Scott's rule. The resulting FWHM after a histogram fit is indicated in the figure for each of the evaluated VOIs before correction, after the $B_{1}^{+}$ correction, and after $B_{1}^{+}$ and $B_{1}^{-}$ correction.

$B_{1}^{+}$ and $B_{1}^{-}$ corrections performed with the $B_{1}$ fields shown in Figure S4 yield improved homogeneity within the phantom (Figure 4). The CV within the whole phantom VOI (Figure 5) decreases from 0.30 before the corrections to 0.20 after the $B_{1}^{+}$ correction and to 0.11 after the $B_{1}^{+}$ and $B_{1}^{-}$ corrections. For the mean value in the whole phantom, higher values in the central region of the uncorrected phantom concentration maps are partially compensated for by the lower apparent concentrations in peripheral regions. In future in vivo segmentations, the evaluated VOIs would be much smaller. For this reason, two smaller VOIs, one placed in the peripheral part of the phantom and one in a central position, were additionally evaluated (see Figure 5A for the VOI placement). Their position is in the lower part of the left lung for the peripheral VOI and in the CSF or IVDs for the central VOI for a volunteer placement as performed in this study. The results are visualized in Figures 5B and 6, and show the under‐ (peripheral cuboid) and overestimation (central cuboid) in the uncorrected data. The deviation from the ground truth value of 35 mm before correction is −5 mm and 22 mm for the peripherally placed cuboid VOI and the centrally placed cuboid VOI, respectively. After the $B_{1}^{+}$ and the $B_{1}^{-}$ correction, the total difference from the ground truth is −1.5 mm and 0.05 mm for the peripherally placed cuboid VOI and the centrally placed cuboid VOI, respectively and thus within one standard deviation from the ground truth (Figure 5B). For the three evaluated VOIs, the FWHM for the in Figure 6 shown histograms decreases due to the corrections. The CV within the peripheral VOI decreases from 0.15 before the corrections to 0.10 after the $B_{1}^{+}$ correction and to 0.06 after the $B_{1}^{+}$ and $B_{1}^{-}$ corrections. For the central small VOI, the CV decreases slightly from 0.043 before the corrections to 0.040 after the $B_{1}^{+}$ correction and to 0.037 after the $B_{1}^{+}$ and $B_{1}^{-}$ corrections.

For the reference vials, the corrections reduce the mean CV for the four reference vials on average from 0.20 before the corrections to 0.13 after the $B_{1}^{+}$ and 0.08 after the $B_{1}^{+}$ and $B_{1}^{-}$ corrections. The R^2^ value for the concentration fits is always high, with 0.97 before the corrections and 0.99 after the $B_{1}^{+}$ and 0.98 after the $B_{1}^{+}$ and $B_{1}^{-}$ corrections.

### 3.2 In vivo measurements

**FIGURE 7:**
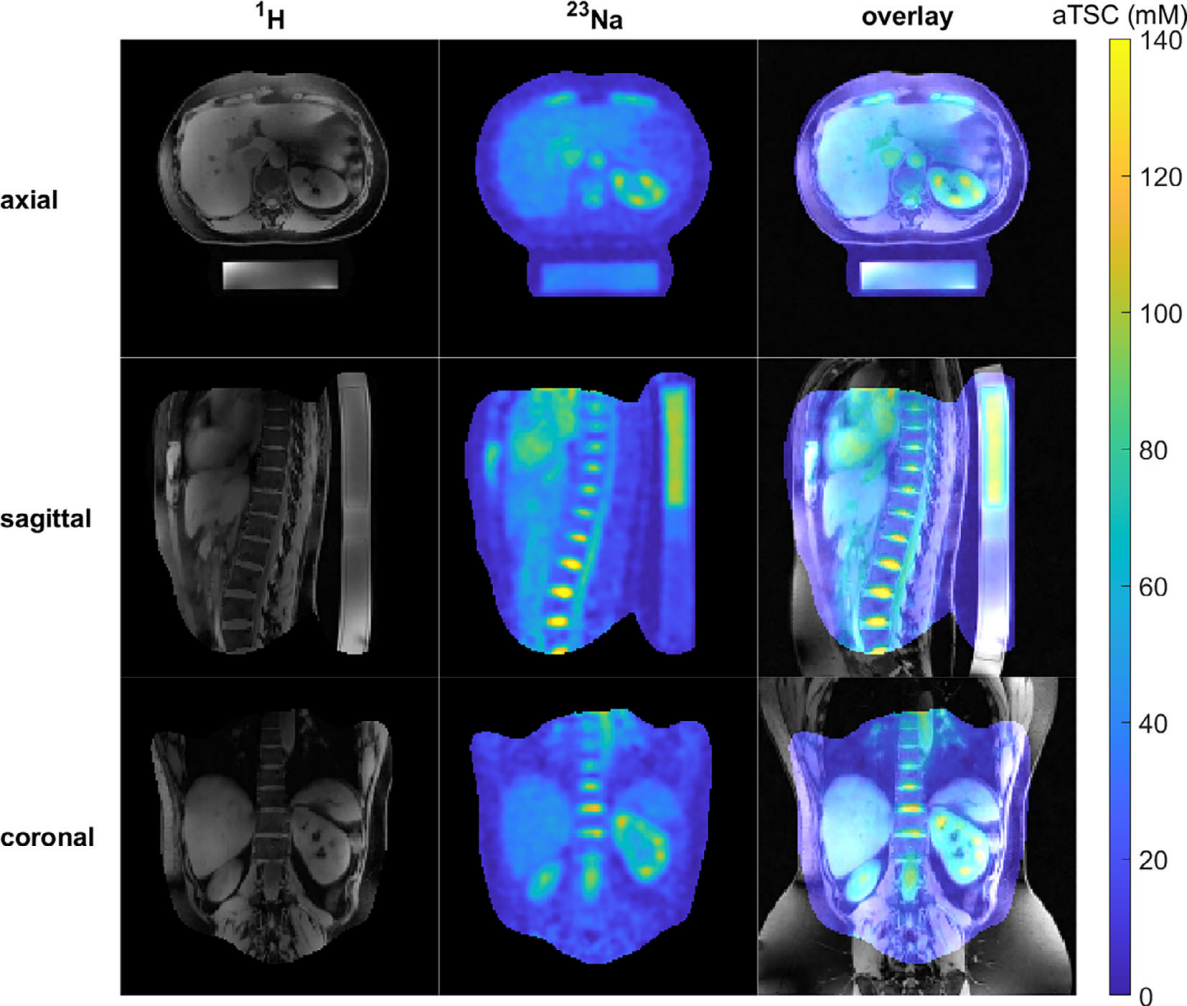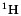 3D GRE‐RPE static pTx images (left), quantitative 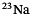 images (middle), and overlay of both (right) for the first volunteer. The 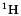 and 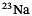 images are both masked with the same mask for easier comparison while the 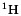 image in the right column is unmasked to show the full FOV.

**FIGURE 8:**
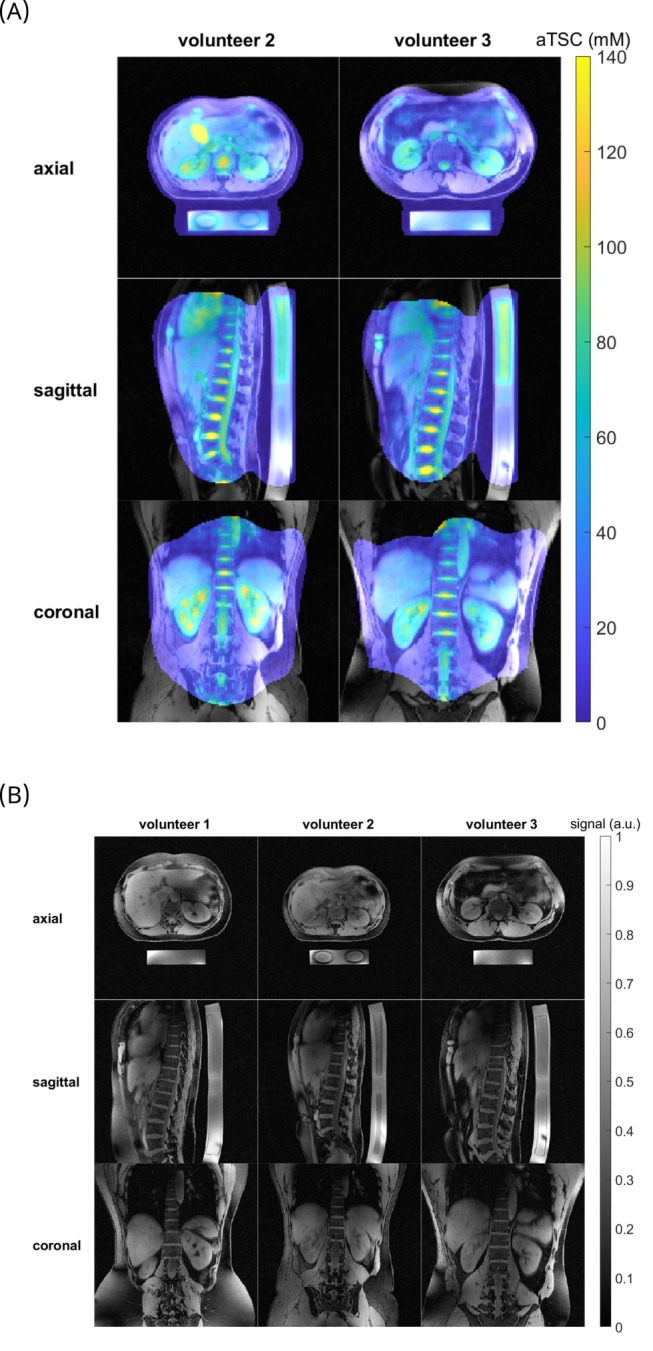
Overlay of 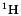 3D GRE‐RPE static pTx images and quantitative 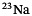 images for volunteer two and three (A) as well as the 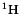 images before interpolation (acquired nominal resolution of (1.25 mm)^3^) for all three volunteers (B).

3D 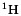 image and corresponding quantitative aTSC map, as well as an overlay of both for one of the volunteers, is shown in Figure 7. Figure 8 shows an overview for the other volunteers that were measured (Figure 8A) and the 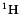 images before interpolation for all volunteers (Figure 8B). It is particularly easy to assess the success of the overlay of the 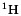 and 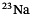 images in the intervertebral discs (IVD), renal, and reference vial areas.

For the reference vials, the corrections improve the mean CV for the four reference vials on average from 0.15 before the corrections to 0.13 after the $B_{1}^{+}$ correction and to 0.09 after the combined $B_{1}^{+}$ and $B_{1}^{-}$ corrections, respectively. Exemplary $B_{1}$ maps for volunteer one are provided in Figure S1. The R^2^ value for the concentration fits was above 0.99 in all cases.

## 4 DISCUSSION

The presented results show the feasibility of full body width quantitative 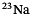 MRI combined with 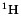 MRI in the human torso with a large z‐coverage at reasonable scan times. The large field‐of‐view of (400 mm)^3^ is beneficial when several organs are of interest.

The high field strength of 7 T yields adequate SNR in an acceptable measurement time for 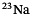 MRI. The high operational frequency of proton MRI at 7 T leads to signal dropouts due to the transmit field inhomogeneity.^35^ For this work, pTx^32^ and TIAMO^33^ were used to improve the homogeneity. As no 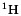 $B_{1}^{+}$ maps were acquired in this study, transmit fields were not optimized for each volunteer and thus a TIAMO shim optimized in a tissue‐simulating phantom was applied to acquire 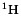 MR data. This resulted in residual 7 T‐related 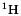 signal dropouts, which were particularly pronounced in the phantom due to the disadvantageous dielectric properties of the NaCl solution within the phantom. Remaining signal dropouts could be located in the region of interest, complicating or impeding image segmentation for certain small structures. In the future, phantom‐ or subject‐specific optimization of the TIAMO shims could further minimize these dropouts. Furthermore, 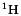 images are not intensity corrected. The 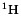 image quality is nevertheless sufficient for segmentation of different central organs and tissue types (e.g., liver, kidney, spleen, gallbladder, cerebrospinal fluid, IVD), which is why an improved 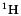 image quality is not necessary here.

Some previous studies investigated 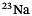 MRI in the torso qualitatively,^20^, ^22^ but without 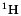 imaging. At 3 T some groups combined the integrated 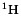 body coil with a 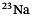 surface coil to investigate the corticomedullary signal gradient in the kidneys qualitatively,^48^, ^49^ and quantitatively^11^ or the prostate quantitatively.^50^ Other studies performed quantitative 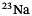 MRI and registered 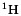 data that was acquired with a different coil setup^14^ or at a different scanner.^12^ As MRI can be performed here with the integrated 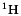 32‐channel pTx body coil and the local 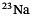 body coil in one examination, a simple overlay of the 3D datasets of the 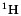 and the quantitative 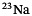 data greatly simplifies the evaluation and allows for the simultaneous quantification in several organs at once. For two examinations with, e.g., two different local RF coils, a different positioning of the subject and physiological state between the 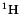 and 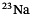 scans could lead to inaccurate image registration and aTSC determination.^1^ Dual‐nuclei coils share this benefit but mostly have a small FOV^16^, ^23^, ^24^, ^51^, ^52^, ^53^ and can thus only investigate a single organ at a time. In other cases, the FOV is bigger, but the acquisition time was kept short by acquiring only 3 slices for 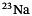, limiting the acquisition on just the IVDs.^28^ While there are other studies that perform whole body 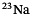 MRI such as Wetterling et al.,^22^ they did not quantify the TSC and did not include 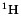 MRI. With our setup, we can investigate the sodium concentration quantitatively and combine it with 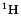 MR in the same position within a large field‐of‐view.

The large distance of the 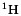 RF coil from the investigated subject in our setup has the disadvantage of a lower receive sensitivity compared to local coils.^54^ Additional 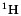 receive coils could improve the SNR, but these would have to be adjusted to the available space in the bore where the 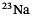 coil is already located. Depending on the structures that are of interest, the limited 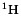 SNR could hamper image segmentation. Nevertheless, the placement of the 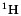 coil behind the scanner bore has the advantage that the coil for the lower SNR nucleus is placed closer to the subject in our setup, whereas other setups have the opposite order.^52^ This leaves enough room for external reference vial placement within the 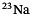 coil when combined with 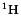 MRI.

The concentration determination with external reference vials, as presented here, has the disadvantage of reducing the space in the 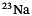 coil, which could exclude some volunteers or patients from the measurement. Additionally, the placement of the vials at the edge of the FOV can lead to quantification errors due to enhanced $B_{0}$ and $B_{1}$ inhomogeneities. These errors can be reduced with proper correction methods. Internal references such as blood^12^ or cerebrospinal fluid^28^, ^55^ are less susceptible to these issues due to their more central position within the coil but face other limitations, such as a limited number of samples, partial volume effects, and potentially inaccurate relaxation times and 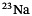 concentrations. They could also show changes in aTSC due to diseases.^56^ For these reasons, external references were used for signal calibration in this study. The reference vials used were chosen to be fairly large to reduce the influence of partial volume effects on the parts used for concentration determination that could otherwise lead to an overestimation of the signal intensities after correction. Due to space restrictions, some previous studies used small external reference vials for correction.^14^, ^16^ Another option for signal calibration is conducting a separate scan with a phantom that replicates the electrical properties of the human torso and includes an appropriate number of reference samples as done in Ouwerkerk et al.^57^ This approach needs to correct for the coil loading to be the same for both the phantom and in vivo measurements, which may be challenging to achieve in some cases.

While Lott et al.^12^ showed a minimal effect of a $B_{0}$ correction on the aTSC, Gerhalter et al.^58^ showed a more significant influence, possibly due to the use of external reference vials with relatively high off‐resonance values compared to the use of blood as an internal reference in Lott et al.^12^ While $B_{0}$ field inhomogeneity for sodium imaging has a relatively small frequency range,^58^ $B_{0}$ effects on the 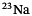 quantification were not considered in this work and implementing this as an additional correction could further enhance the quantification accuracy. The achieved homogeneity within the phantom and reference vials was nevertheless high, and quantification worked well for the whole phantom as well as smaller volumes inside the phantom.

We measure $B_{1}^{+}$ and simulate $B_{1}^{-}$ for 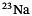 image corrections for the utilized setup, giving us an advantage over studies that only measure $B_{1}^{+}$ and assume $B_{1}^{-}=B_{1}^{+}$ ^59^, ^60^ where this assumption might not hold true^61^ or measure $B_{1}^{+}$ in a phantom for $B_{1}^{+}$ and $B_{1}^{-}$ correction.^13^, ^16^ Here, it is an advantage that we have detailed knowledge of the custom‐built RF hardware^21^, ^35^ and are thus able to perform these simulations. Future studies could investigate the influence of universal $B_{1}^{-}$ corrections for this setup on the determined aTSC of different abdominal structures to evaluate the necessity of an individualized $B_{1}^{-}$ correction to determine an accurate aTSC.

We used a free‐breathing acquisition without respiratory sorting by applying sequences that are less prone to artifacts due to respiratory motion for both 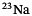 and 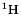 measurements. Simulations accessing subject motion combined with retrospective respiratory sorting of image data could investigate the influence on the segmentation and aTSC accuracy when taking respiratory motion into account.

As the data for the current study are acquired in free breathing, respiratory motion blurs the reconstructed images. A potential future implementation to reduce measurement time and account for motion in the 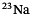 data could be XD‐GRASP,^62^ which uses compressed sensing^63^ on data sorted into multiple motion states. Another way to reduce the scan time and thus some of the motion (e.g., due to digestion or muscle relaxation) would be to interleave the 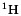 and 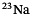 acquisitions,^64^ using the long $T_{R}$ of sodium for proton data acquisition, as, e.g., done in Wilferth et al.^65^ and de Bruin et al.^66^ This is currently not easily achievable with our setup due to hardware and software limitations. Reducing the overall scan time further will open up the possibility to measure a wider range of volunteers and patients.

In this work, the focus was to gather data that could give an accurate estimation of the TSC with our setup (e.g., by applying long $T_{R}$). The influence on the aTSC when reducing the scan time could be investigated in the future. The effect of undersampling the 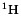 images on the segmentation and thus aTSC could also be examined to potentially further lower the measurement time.

Understanding the aTSC in a large FOV could, e.g., assist in monitoring the treatment progress of chemotherapy patients with widespread metastases.^22^ Another possible application could be the investigation of the cardiorenal syndrome.^29^ The simultaneous acquisition of the kidneys and heart could save scan time and help to gain a better understanding of the interdependence of both organs. Additionally, a large FOV allows investigation of body regions that were not investigated before, such as the pancreas and stomach, without performing an additional scan.

## 5 CONCLUSIONS

In this work, the feasibility of combined 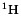 and quantitative 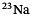 imaging at 7 T in a large field‐of‐view in the human torso under free breathing is shown in three healthy volunteers with an acquisition time of about 47min. Reconstruction of both 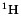 and 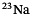 data in the same position allows for a simple overlay of the two 3D datasets and detailed assessment of a large number of organs and tissues in one examination. Using the presented reference vial setup combined with the performed $T_{1}$, $B_{1}^{+}$ and $B_{1}^{-}$ corrections, the ground truth concentration value in a phantom could be confirmed in 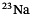 concentration determinations in a large as well as in smaller volumes.

The presented workflow provides the basis for accurate measurement of the aTSC in multiple abdominal organs simultaneously, allowing the study of the interplay between multiple organs or the detection of changes due to physiological processes in multiple regions of the body.

## CONFLICT OF INTEREST STATEMENT

The authors declare no potential conflict of interest.

## FUNDING INFORMATION

None reported.

## Supporting information

The following supporting information is available as part of the online article:
**Figure S1.** Dimensions and positioning of the reference vial VOIs.
**Figure S2.** Alignment of 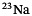 and 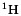 images in the phantom for the anterior‐posterior direction in a transversal slice.
**Figure S3.** Alignment of 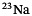 and 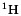 images in the phantom for the cranial‐caudal direction in a sagittal slice.
**Figure S4.** Measured $B_{1}^{+}$ maps and simulated $B_{1}^{-}$ maps for phantom data.
**Figure S5.** Measured $B_{1}^{+}$ maps and simulated $B_{1}^{-}$ maps for in vivo data.
