# Supplementary material for: Quantitative abdominal sodium MRI combined with 32‐channel proton pTx MRI at 7 Tesla in a large field‐of‐view
Source: Magn Reson Med. 2025 Jun 22;94(5):1930–45. doi: 10.1002/mrm.30605 (PMC12393210; doi:10.1002/mrm.30605)
Supplement: Supplementary file 1 — The following supporting information is available as part of the online article: Figure S1. Dimensions and positioning of the reference vial VOIs. Figure S2. Alignment of and images in the phantom for the anterior‐posterior direction in a transversal slice. Figure S3. Alignment of and images in the phantom for the cranial‐caudal direction in a sagittal slice. Figure S4. Measured B1+ maps and simulated B1− maps for phantom data. Figure S5. Measured B1+ maps and simulated B1− maps for in vivo data. [file MRM-94-1930-s001.pdf]

Supporting Information

axial

coronal

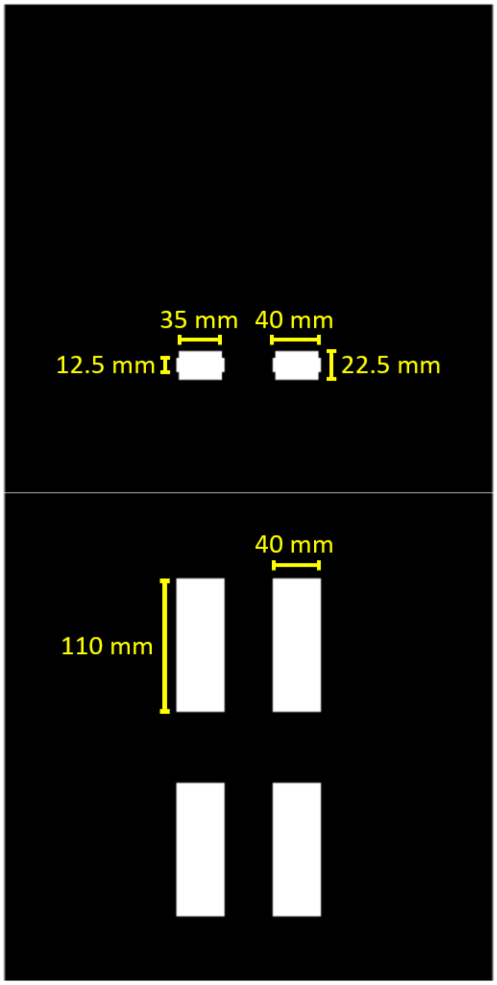

Figure S1: Vial placement and shape annotated with the respective size. All reference vial masks have the same size.

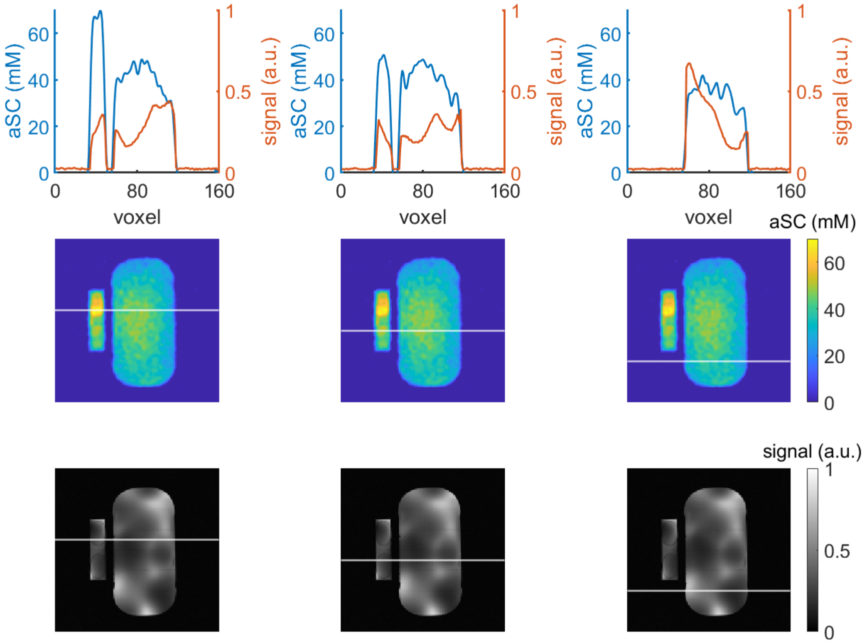

Figure S2: Alignment of ^{23}Na and ^{1}H images in the phantom for the anterior-posterior direction in a transversal slice. The line plot in the first row displays the values in the uncorrected ^{23}Na (blue line) and ^{1}H (orange line) MRI along the white line in the second and third row, respectively. For each plotted line through the phantom, the upper border of the phantom and reference vials in the ^{23}Na image correspond well to those in the ^{1}H image. In the third column, where the values are only in the phantom, the images are aligned well.

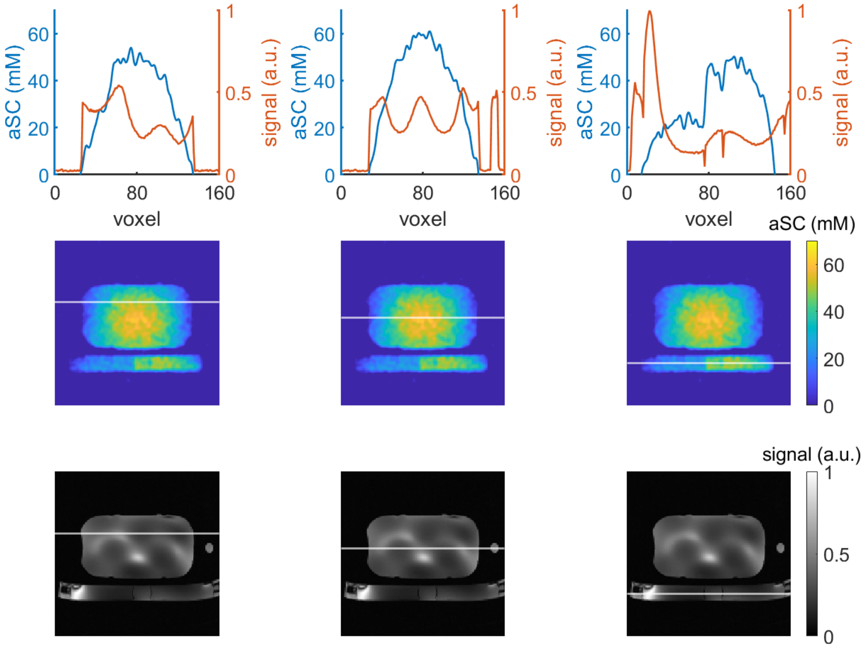

Figure S3: Alignment of ^{23}Na and ^{1}H images in the phantom for the cranial-caudal direction in a sagittal slice. The line plot in the first row displays the values in the uncorrected ^{23}Na (blue line) and ^{1}H (orange line) MRI along the white line in the second and third row, respectively. For each plotted line through the phantom, the ^{23}Na and ^{1}H image are well aligned. In the third column, where values within the reference vial setup are plotted, the signal decrease towards the edges in the ^{23}Na image leads to a line plot mismatch. For high $|z|$-values, gradient non-linearity artifacts can be observed in the ^{1}H MR images.

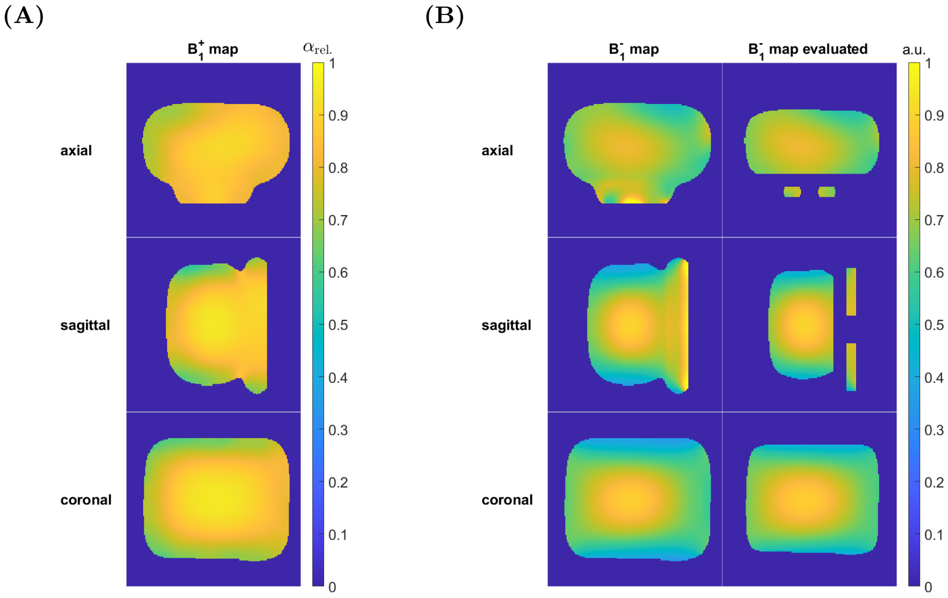

Figure S4: Measured B_1^+ map (A) and setup specific simulated B_1^- map (B) for the phantom measurement. The relative B_1^+ map was calculated as $\alpha_{\text{rel.}} = \alpha/\alpha_0$. For the B_1^- map, the evaluated volumes of interest (phantom and reference vials) are shown in the second column.

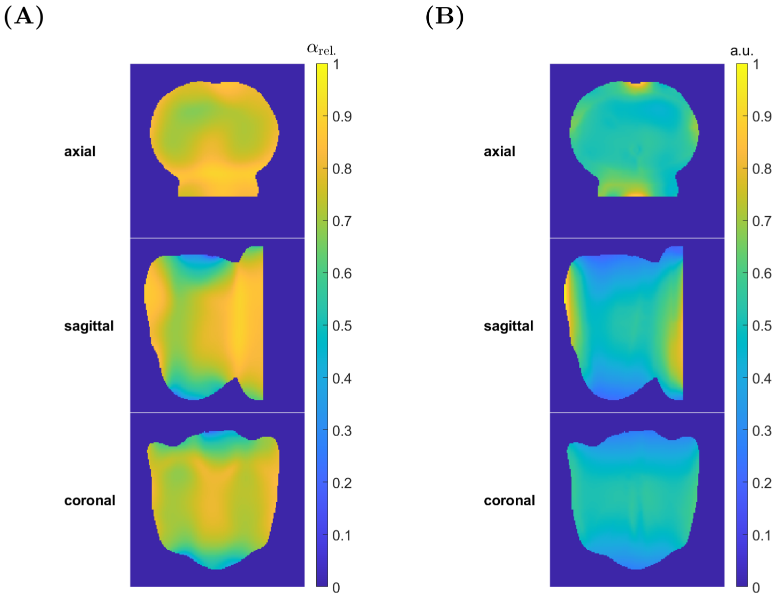

Figure S5: Measured B_1^+ map (A) and setup specific simulated B_1^- map (B) for the in vivo measurement of volunteer one. The relative B_1^+ map was calculated as $\alpha_{\text{rel.}} = {}^{\alpha}\!/\!_{\alpha_0}$.
